# Elements of Chemistry, Theoretical and Practical

**Published:** 1845-09

**Authors:** 


*Elements of Chemistry, Theoretical and Practical.* By GEORGE FOWNES, Ph. D., Chemical Lecturer in the Middlesex Hospital School, and to the Pharmaceutical Society of Great Britain. With numerous illustrations. Edited, with additions, by ROBERT BRIDGES, M. D., Professor of General and Pharmaceutical Chemistry in the Philadelphia College of Pharmacy, &c. &c. 8vo. pp. 460. Lea and Blanchard, Philadelphia, 1845.

The design of the author in the preparation of this volume appears to have been to present to the student of Chemistry, in a compact and inexpensive, but intelligible form, "an outline of the general principles of that science, and a history of the more important among the very numerous bodies which chemical investigations have made known to us." The author modestly assures us that "the work has no pretensions to be considered a complete treatise on the subject, but is intended to serve as an introduction to the larger and more comprehensive works in our own language and in those of the Continent." Notwithstanding this announcement, we are presented with a work, not only comprehensive as regards general principles, but full of practical details of the working processes of the scientific laboratory; and in addition, it contains numerous wood engravings, showing the most useful forms of apparatus, with their adjustments and methods of use.

What adds materially to the value of this work is the view which it contains of our present knowledge of organic chemistry. To the ultimate analysis of organic bodies the author has devoted, indeed, considerable space, and has exhibited the subject in as clear and intelligible a point of view, probably, as it is susceptible of, in its present progressive state. So great is the labor now employed on this intricate branch of chemistry, and so important and numerous have been the discoveries of Liebig and others, that the old land-marks are removed, and an entirely new field opened to our view—a field teeming with the brightest objects for the contemplation of the chemical philosopher.

The original work having been full and complete, as far as the limits of such a volume would permit, and on every point brought up to the date of its publication (in September last,) the task of the editor has been to add any important matter which appeared since, and to correct such typographical errors as had escaped the author. That this task has been well and ably performed, the known zeal and competency of Dr. Bridges afford a sufficient guarantee. In mechanical execution, the present edition is to be commended in every thing but the quality of the paper, which is not in keeping with the typography and excellent wood engravings.

The volume is of a very convenient size for a text-book for medical students, and is in all other respects well adapted to their wants; nor will it be found less suitable as a book of reference for physicians and practical chemists, who may desire to keep pace with the rapid advancement of the science.

